# Increasing self-other bodily overlap increases sensorimotor resonance to others’ pain

**DOI:** 10.3758/s13415-019-00724-0

**Published:** 2019-06-12

**Authors:** Igor Riečanský, Lukas L. Lengersdorff, Daniela M. Pfabigan, Claus Lamm

**Affiliations:** 1 Social, Cognitive and Affective Neuroscience Unit, Department of Basic Psychological Research and Research Methods, Faculty of Psychology, University of Vienna, Liebiggasse 5, A-1010 Vienna, Austria; 2 Department of Behavioural Neuroscience, Institute of Normal and Pathological Physiology, Centre of Experimental Medicine, Slovak Academy of Sciences, Sienkiewiczova 1, Bratislava, 81371 Slovakia

**Keywords:** Empathy, Racial bias, Body ownership, Self, Self-other distinction, Pain

## Abstract

Empathy for another person’s pain and feeling pain oneself seem to be accompanied by similar or shared neural responses. Such shared responses could be achieved by mapping the bodily states of others onto our own bodily representations. We investigated whether sensorimotor neural responses to the pain of others are increased when experimentally reducing perceived bodily distinction between the self and the other. Healthy adult participants watched video clips of the hands of ethnic ingroup or outgroup members being painfully penetrated by a needle syringe or touched by a cotton swab. Manipulating the video presentation to create a visuospatial overlap between the observer’s and the target’s hand increased the perceived bodily self-attribution of the target’s hand. For both ingroup and outgroup targets, this resulted in increased neural responses to the painful injections (compared with nonpainful contacts), as indexed by desynchronizations of central mu and beta scalp rhythms recorded using electroencephalography. Furthermore, these empathy-related neural activations were stronger in participants who reported stronger bodily self-attribution of the other person’s hand. Our findings provide further evidence that empathy for pain engages sensorimotor resonance mechanisms. They also indicate that reducing bodily self-other distinction may increase such resonance for ingroup as well as outgroup targets.

**Electronic supplementary material:**

The online version of this article (10.3758/s13415-019-00724-0) contains supplementary material, which is available to authorized users.

## Introduction

Empathy is an important social cognitive capacity that enables us to share and understand the feelings of other people (Coplan & Goldie, 2011). In recent years, considerable progress has been made in identifying the neural mechanisms of empathy. Research using functional neuroimaging and electrophysiological methods in humans has shown that observing other individuals in an emotional state results in similar brain activations as being in the same emotional state oneself (Coll & Jackson, 2016; Lamm et al., 2017). Such so-called shared neural activations have been identified in brain regions involved in affective, sensory, and motor processing and are considered to be crucial for empathy (Rütgen et al. 2015, 2018; but see Krishnan et al. 2016). This has been demonstrated consistently using the so-called empathy for pain paradigm: being in pain and seeing pain inflicted on others both result in increased activations in several brain regions, including insular, cingulate, and sensorimotor cortex (Betti & Aglioti, 2016; Bufalari & Ionta, 2013; Keysers et al. 2010; Lamm et al. 2011, 2016, 2017; Zaki et al. 2016).

Social cognition heavily draws on bodily self-awareness, and recent experimental investigations confirm that such processes affect how we perceive and act on social signals (Maister & Tsakiris, 2016). For example, the so-called rubber hand illusion can be induced by touching a participant's hand while they observe an artificial (rubber) hand being touched in synchrony with their own. This leads to bodily self-attribution of the rubber hand, i.e., the impression that the artificial hand is part of the person’s own body (Botvinick & Cohen, 1998). Similar illusions can be elicited for other body parts, including the whole body (Costantini, 2014; Kilteni et al. 2015). A number of studies have shown that such manipulations of bodily self-awareness affect the processing of social information. For instance, when the body ownership illusion was applied to a face, participants reported more positive attitudes toward the other person (Paladino, Mazzurega, Pavani, & Schubert, 2010; Tajadura-Jiménez, Longo, Coleman, & Tsakiris, 2012). There also is consistent evidence that inducing illusory ownership of an outgroup body has the potential to reduce bias against that outgroup (Banakou et al. 2016; Farmer et al. 2014; Hasler et al. 2017; Maister et al. 2013; Peck et al. 2013; Maister et al. 2015). This opens avenues for interventions against such biases, which still seem to pervade our society.

These studies indicate that decreasing the bodily boundary between self and other results in increased prosocial behaviors and attitudes toward that person, or even the social group to which he or she pertains. Because empathy is one of the factors driving prosocial behavior (Davis, 2015; Lamm et al. 2017), these investigations suggest that decreasing the bodily boundary between self and other could increase empathic responses. This hypothesis is indirectly supported by a number of findings. First, trait empathy is positively associated with susceptibility to bodily illusions (Asai, Mao, Sugimori, & Tanno, 2011; Farmer, Tajadura-Jiménez, & Tsakiris, 2012; Seiryte & Rusconi, 2015). Second, higher empathy has been reported in individuals with mirror-touch or mirror-pain synesthesia, who experience tactile sensations or pain when they see someone else being touched or painfully injured (Banissy & Ward, 2007; Osborn & Derbyshire, 2010). Notably, such synesthesia conditions can be construed as a condition of loosened bodily boundaries between self and other (Ward & Banissy, 2015). Third, illusions of ownership of other's hand were found to modulate excitability of primary motor cortex when observing that hand being painfully stimulated (Avenanti, Bueti, Galati, & Aglioti, 2005; Bucchioni et al. 2016; De Coster, Andres, & Brass, 2014).

Electroencephalographic (EEG) and magnetoencephalographic (MEG) studies investigating the modulation of oscillatory mu (7–12 Hz) and beta (13–30 Hz) rhythms recorded over the central cortex provide an important source of evidence for sensorimotor activations in the processing of social signals. The terms *event-related desynchronization* (ERD) and *synchronization* (ERS) are used, respectively, to denote event-related suppression and enhancement of the EEG/MEG oscillatory activity (Pfurtscheller & da Silva, 2005). ERD of central rhythms occurs during both execution and observation of movements (Avanzini et al. 2012; Babiloni et al. 2002; Woodruff & Klein, 2013; Woodruff, Martin, & Bilyk, 2011), as well as during both somatosensory stimulation and observation of bodily contacts (Cheyne et al. 2003; Whitmarsh, Nieuwenhuis, Barendregt, & Jensen, 2011). Several recent studies identified modulations of sensorimotor rhythms in association with empathy-related processing. In particular, it has been reported that seeing other individuals in painful relative to nonpainful situations results in a stronger suppression of sensorimotor rhythms (Betti & Aglioti, 2016; Chen, Yang, & Cheng, 2012; Cheng, Chen, & Decety, 2014; Fabi & Leuthold 2017, 2018; Grice-Jackson, Critchley, Banissy, & Ward, 2017; Hoenen, Lübke, & Pause 2015; Perry, Bentin, Bartal, Lamm, & Decety, 2010; Whitmarsh et al. 2011; Yang, Decety, Lee, Chen, & Cheng, 2009). For instance, in one of our own recent studies (Riečanský, Paul, Kölble, Stieger, & Lamm, 2015), central beta ERD was significantly stronger when participants observed videos depicting painful needle injections compared with nonpainful contacts by a cotton swab. Interestingly, these empathy-related neural responses were only elicited for ethnic ingroup targets, indicating the presence of an ethnic intergroup bias in empathy, on the neural level. Moreover, they were reliably present only in the beta but not in the mu band.

Combining these two research strands, the current study set out to test the hypothesis that bodily self-awareness affects empathy-related sensorimotor activations. In particular, we expected that weakening the bodily boundary with another individual would enhance empathy-related sensorimotor activation and reduce the ethnicity ingroup bias in such activation that we had observed previously. To assess this research question, we followed the experimental design of our previous study but incorporated a decisive change in how the visual stimuli of the previous experiment were presented: the right hands of the targets were displayed on a flat screen placed directly over the participant's own right hand (De Coster et al. 2013; Höfle et al. 2012, for similar approaches). With this manipulation, we intended to increase bodily overlap with the target and predicted that this would increase ERD of the sensorimotor rhythms when seeing painful actions toward the targets. Furthermore, we expected this manipulation to increase empathy-related neural responses toward the ethnic outgroup targets to an extent that it could act as a possible remedy against the previously observed ethnic ingroup bias in empathy.

Our study consisted of two experiments. First, we performed a behavioral experiment to test whether presenting a hand on a screen placed over the participant's hand (*overlap presentation*) evoked increased perceptions of bodily self-attribution of the target's hand compared with presenting the hand on a monitor placed upright in front of the subjects (*no-overlap presentation*). In a subsequent EEG experiment, we investigated if this kind of presentation enhanced neural responses to painful stimulation inflicted on the depicted hand. We collected and analyzed EEG data using the overlap setup in a novel independent sample and compared them to the data from our earlier study, which had employed the no-overlap setup (Riečanský et al., 2015). We deliberately decided against a within-subjects design, i.e., administering both the overlap and the no-overlap conditions in this novel sample due to expected substantial and systematic carry-over effects from the overlap to the nonoverlap condition, which did not allow us to counterbalance the order of the two conditions across participants.

## Behavioral experiment – validation of the overlap stimuli presentation

### Methods

#### Participants

Participants were white Caucasian healthy adult volunteers with right-handedness preference (Oldfield, 1971) and normal or corrected-to-normal vision. Participants with history of neurological or psychiatric disorders, traumatic head injury, regular medication use, or abuse of psychotropic drugs were excluded from participation. Participants were randomly assigned to two groups, which differed in the presentation setup (overlap vs. no-overlap, see section 2.1.2). The "overlap group" consisted of 20 participants (11 females, 9 males, mean age (SD) = 20.3 (2.0) years), while 22 participants were included in the "no-overlap group" (11 females, 11 males, mean age (SD) = 20.6 (1.5) years). All participants signed written, informed consent and received course credit for study participation. This as well as the following EEG experiment were conducted in line with regulations by the local ethics committee and the ethical standards declared in the Declaration of Helsinki.

#### Presentation setup

**Fig 1.**
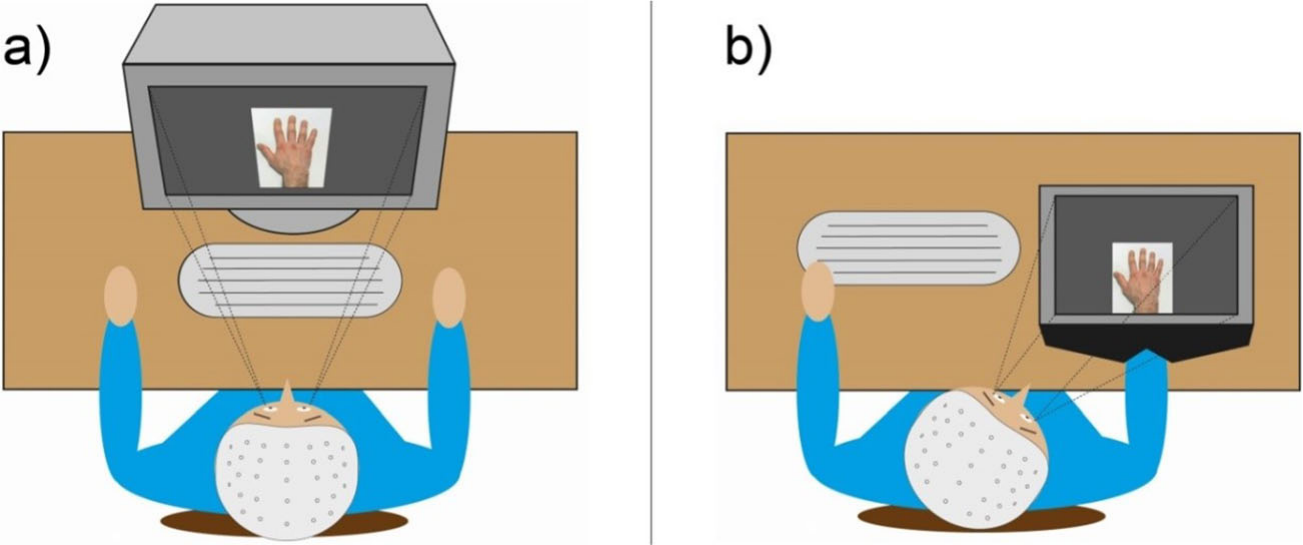
Schematic depiction of the experimental settings used in the behavioral experiment. (a) No-overlap projection, (b) overlap projection

The experiments were conducted in a darkened, sound-insulated EEG recording chamber. Figure 1 provides a schematic depiction of the presentation setups. In the *no-overlap* setup, stimuli were presented on a monitor (19-inch, CRT, Sony GDM-F520) placed upright in front of the subjects (distance from eyes to the monitor was approximately 70 cm). To keep proprioceptive input equal, the participants were asked to lay their hand onto the tabletop similar to participants in the overlap group. In the *overlap setup*, stimuli were presented on a flat screen monitor (Elo 1529L, 15 inch), which was positioned horizontally on top of a box with an open front bottom panel, forming an opening through which the hand could be inserted. The participants placed their right hand into the box, then the position of the box was adjusted so that the hand on the screen overlapped with the participant’s own hand. To eliminate visual distractors, the box was covered with a black cloth, but excluding the monitor.

#### Stimuli

**Fig 2.**
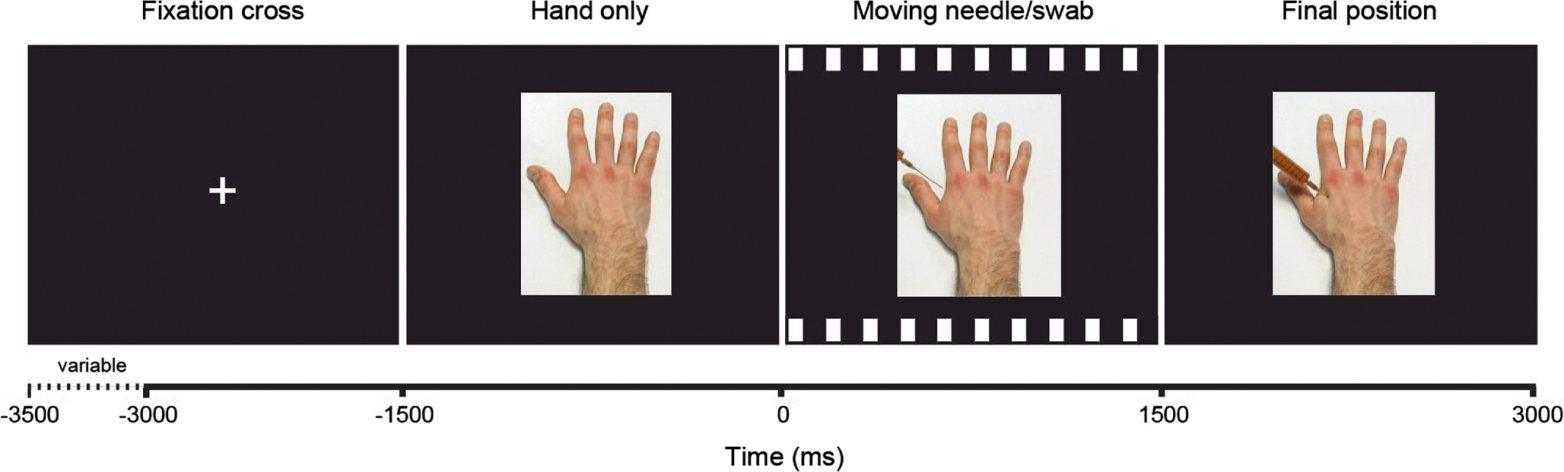
Schematic display of the visual stimuli and their timing. The trial sequence began with presenting a fixation cross (duration varied between 1,500 and 2,000 ms), followed by a static display of a hand (duration = 1,500 ms). This was followed by the video showing the action of hand treatment (i.e., motion of a needle syringe or a cotton swab, duration = 1,500 ms). After the needle syringe or the cotton swab had reached their final position, a static display of the last frame of the video was shown (duration = 1,500 ms). Next trial followed automatically without delay

We used short videos developed by Avenanti et al. (2010) depicting the following conditions: a needle syringe penetrating a white hand (Ingroup Pain) or a black hand (Outgroup Pain); a cotton swab touching a white hand (Ingroup No-pain) or a black hand (Outgroup No-pain). All videos depicted right hands in first-person perspective. For each condition, there were three different videos with slight variations in the appearance (size of syringe and color of swab). Videos were presented in four blocks of 12 trials each, with every block only containing videos of one condition (i.e., each video was presented 4 times), resulting in a total of 48 trials. The sequence of trials within blocks and the order of blocks were randomized. Figure 2 illustrates the sequence and the timing of stimuli within one trial – please refer to the more detailed description in the figure caption. Stimuli ratings were performed after each block. Stimuli were presented and responses were collected using E-Prime 2.0 (Psychology Software Tools, Inc., Sharpsburg, PA).

#### Ratings of perceived bodily self-attribution

Following Longo et al. (2008), after each block of stimuli presentation participants rated three aspects of perceived bodily self-attribution of the target hand (in the following order): (i) *ownership*: how much the hand on the screen was perceived as one's own hand, (ii) *agency*: how much participants felt that they could control the hand on the screen, (iii) *location*: how much one's own hand was perceived to be at the same location as the hand on the screen, and a 7-point Likert scale was used with values ranging from "not at all" (0) to "very strongly" (6).

#### Statistical analysis

PASW Statistics 18 (SPSS Inc., Chicago, IL) was used for statistical analyses. The statistical analyses were performed under the framework of the general linear model (GLM) using full-factorial design, fixed effects, and SPSS’s Type III sum-of-squares methods. Significance level for all tests was α = 0.05. A four-way mixed-design analysis of variance (ANOVA) with the between-subjects factor *Setup* (overlap/no-overlap) and within-subjects factors *Ethnicity* (ingroup/outgroup), *Treatment* (painful/nonpainful), and *Dimension* of perceived bodily self-attribution of the target hand (ownership/location/agency or ownership/location) was calculated.

### Results

**Fig 3.**
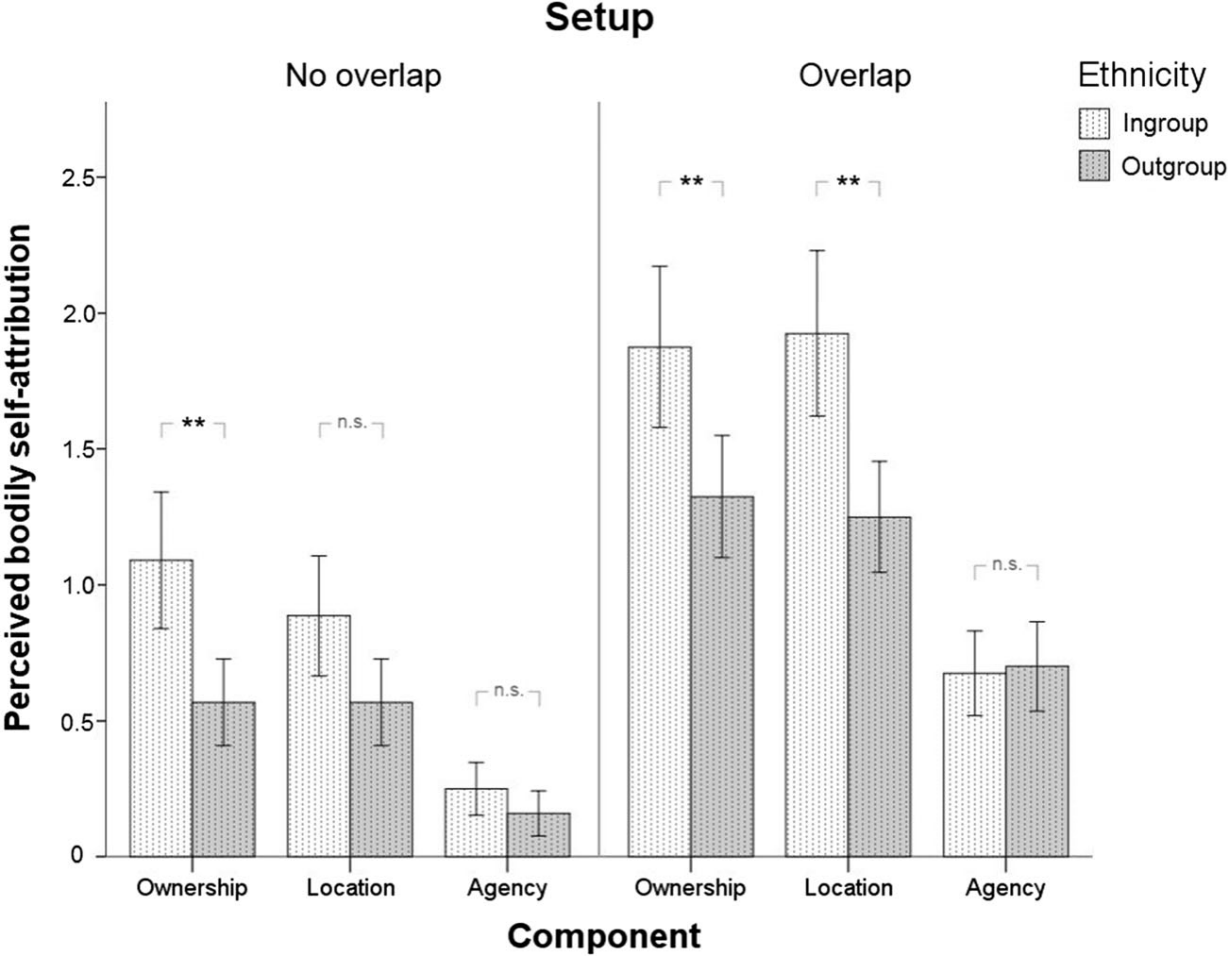
Ratings of perceived bodily self-attribution of the target hand (mean ± standard error of the mean) in the no-overlap setup (n = 20) and the overlap setup (n = 22) plotted separately for ingroup (light bars) and outgroup target hands (dark bars)

Ratings of perceived bodily self-attribution of the depicted hand were significantly higher in the overlap setup compared with the no-overlap setup for both ingroup and outgroup hands (Figure 3, Setup: *F*_*1,40*_ = 9.443, *p* = 0.004, *η*^*2*^_*p*_ = 0.191, all interactions involving Setup were not significant, all *p* ≥ 0.164, for a complete report of the statistical tests see Supplementary Table 1a). Treatment of hands (i.e., observing painful needle injections vs. nonpainful touch of a cotton swab) had no influence on the ratings (all effects including treatment were not significant, all *p* ≥ 0.129). Ratings of Ownership and Location were overall higher for ingroup than outgroup hands (Ethnicity x Dimension: *F*_*2,80*_ = 7.714, *p* = 0.001, *η*^*2*^_*p*_ = 0.162; Ethnicity effect for Ownership*: p* = 0.001; Ethnicity effect for Location: *p* = 0.003). The ratings of Agency were not influenced by the hand’s ethnicity (Ethnicity effect for Agency: *p* = 0.689), but they were much lower than the ratings of Ownership and Location (Dimension: *F*_*2,80*_ = 38.178, *p* < 0.001, *η*^*2*^_*p*_ = 0.488). When the ratings of Agency were excluded from the analysis, the main effect of Dimension as well as the Ethnicity x Dimension interaction were not statistically significant (Supplementary Table 1b). Due to much lower efficacy of our setting to induce the sense of agency over the displayed hand we did not include ratings of this dimension in the subsequent EEG study.

## EEG experiment

### Methods

#### Participants

Thirty volunteers participated in the EEG experiment. None of them had participated in the behavioral experiment. Participants were white Caucasian healthy adult volunteers with right-handedness preference (Oldfield, 1971) and normal or corrected-to-normal vision. Exclusion criteria were the same as in the behavioral study (see Section 2.1.1). The program G*power (Faul et al., 2007) was used to estimate the sample size to detect with a statistical power of 0.80 an effect of *d* = 0.46 (the effect size of the ethnicity bias in empathy-related beta rhythm ERD in our previous study, *N* = 36, Riečanský et al., 2015). One participant was excluded from the sample due to excessive artifacts in the EEG recordings. The final sample thus consisted of 29 participants (16 females and 13 males, mean age (SD) = 24.5 (3.9) years).

#### Trait measures

Trait measures were assessed after the EEG experiment and stimuli-related ratings. The implicit association test (IAT) was used to assess implicit ethnic stereotypes (Greenwald, McGhee, & Schwartz, 1998). Participants categorized stimuli as belonging to the categories *good* or *bad* (words) and *black* or *white* (faces). Based on response latencies, the D-index was computed as a measure of implicit ethnicity bias according to the algorithm described by Greenwald et al. (2003).

To assess ethnic attitudes of study participants, we employed the Attitudes Towards Blacks (ATB) scale (Brigham, 1993). This questionnaire assesses attitudes towards black people in relation to various social issues, such as urban crime, interracial marriage, or racial integration in schools, businesses, and residences.

Dispositional empathy was assessed by using the German version of the Interpersonal Reactivity Index (IRI; Davis, 1983; Paulus, 2009), a questionnaire measuring four aspects of empathy: *perspective taking*, the ability to spontaneously take the perspective of others and to see things from their point of view; *fantasy,* the tendency to identify with other persons; *empathic concern*, addressing feelings of concern toward others; and *personal distress*, assessing feelings of distress when observing others in need.

#### Stimuli

The same stimuli as in the behavioral experiment and in our previous study were used (see Section 2.1.2). For each of the four experimental conditions (Ingroup Pain, Outgroup Pain, Ingroup No-pain, Outgroup No-pain), 60 trials were presented resulting in a total of 240 trials, which were grouped into 20 blocks of 12 trials. Trials of each condition were presented in a randomized order. This protocol precisely matched our previous study (Riečanský et al. 2015). In contrast to that study, however, the stimuli were presented using the overlap setup described in Section 2.1.3.

#### Stimuli-related ratings: state empathy and perceived bodily self-attribution

Stimuli-related ratings were performed immediately after the collection of EEG data. Each video used in the EEG experiment was presented one time to rate state empathy and a second time to rate perceived self-attribution of the target hand. The videos were presented in random order. The ratings of state empathy included (i) target-related *painfulness*: how painful the intervention depicted on the videos was for the target, and (ii) observer-related *unpleasantness*: how unpleasant their own feelings were when watching the stimuli. Perceived bodily self-attribution of the target hand included the rating of *ownership* and *location* (in this order, for details see Section 2.1.4). The ratings of agency were not collected since the behavioral experiment had shown that this aspect of the perceived self-attribution was very weak in the behavioral experiment, see Section 2.2). For all ratings, a 7-point Likert scale was used with values ranging from "not at all" (0) to "very strongly" (6). State empathy ratings for no-pain videos were not analyzed, because the values were close to zero.

#### EEG recording and processing

**Fig 4.**
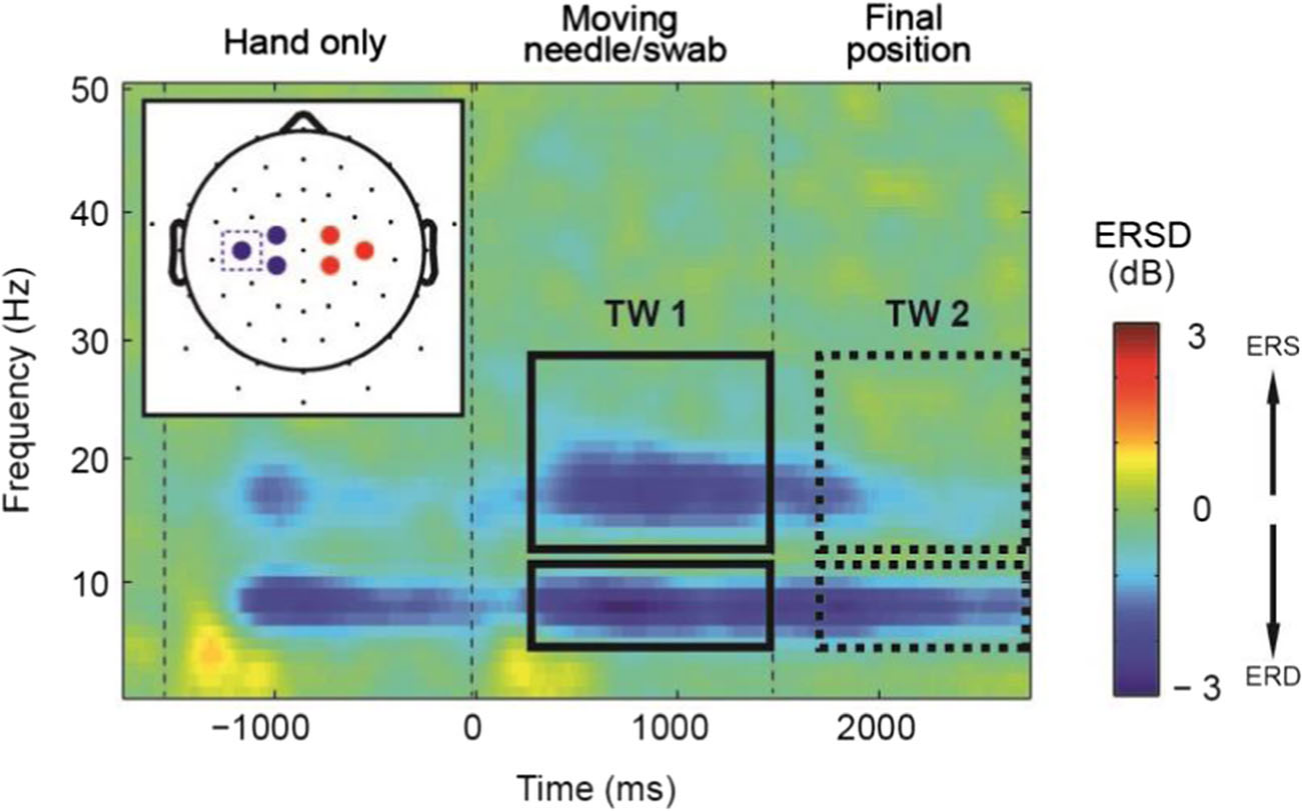
Dynamics of sensorimotor oscillations during observation of the videos. Inserted is a schematic drawing of head depicting positions of EEG sensors (small black dots) and sensors selected for signal analysis (blue circles: left ROI, red circles: right ROI). Mean ERSD (n = 29) in the Ingroup Pain condition is plotted for one sensor overlying left sensorimotor cortex (position C3 of the international 10-20 system, see dashed rectangle in the inserted head plot). The pattern of spectral changes over time at other sensors was similar. Rectangles depict windows for analysis of experimental effects (time window 1: treatment action, time window 2: treatment endpoint)

Collection and processing of EEG data followed exactly the same procedures and methods of our previous study, and we refer the readers to our previous publication for specific details (Riečanský et al., 2015). Briefly, EEG was recorded from 59 equidistantly positioned electrodes mounted on an elastic cap (montage M10, Easycap, Germany). After initial signal processing and artifact removal using the EEGLAB toolbox (Delorme & Makeig, 2004), the EEG signals were transformed to reference-free scalp current source density (CSD) to eliminate volume-conducted contributions from distant regions and hence signals likely not originating in sensorimotor cortex (Kayser, 2009). Sensors overlying left and right sensorimotor cortex were selected to represent regions of interest (ROIs, see insert in Figure 4). In these channels (for each sensor separately), event-related spectral power modulation (also termed event-related spectral perturbation or event-related synchronization/desynchronization, ERSD) was assessed for each subject and experimental condition (Delorme & Makeig, 2004) with respect to prestimulus baseline period (from −2,000 ms to −1,500 ms; Figure 1). Mean ERSD was then calculated within the frequency bands 7-12 Hz (alpha/mu) and 13-30 Hz (beta), within each ROI.

#### Statistical analysis

PASW Statistics 18 (SPSS Inc., Chicago, IL), MATLAB (The Mathworks, Massachusetts, MA), and R (R Core Team, 2016) were used for statistical analyses. Statistical analyses largely followed procedures of our previous work (Riečanský et al., 2015) and were performed under the framework of the general linear model (GLM) using full-factorial design, fixed effects, and SPSS’s Type III sum-of-squares methods. Significance level for all tests was α = 0.05. To analyze perceived self-attribution, a three-way within-subjects ANOVA was used with factors *Ethnicity* (ingroup/outgroup), *Treatment* (painful/nonpainful) and *Dimension* (ownership/location). Ethnicity effects on state empathy ratings were analyzed using *t* tests for correlated samples. Ratings of state empathy were compared between the studies using a two-way mixed-design ANOVA with between-subjects factor *Setup* (no-overlap/overlap) and *Ethnicity* (ingroup/outgroup). ERSD data were analyzed separately for the mu and the beta band with a three-way within-subjects ANOVA with factors *Ethnicity* (ingroup/outgroup), *Treatment* (painful/nonpainful), and *Hemisphere* (left/right)*.* For these ANOVAs, to prevent a bias in statistical inference due to outliers and in agreement with our previous study (Riečanský et al., 2015), values outside the interval 〈*Q*1 − 2 ∗ *IQR*, *Q*3 + 2 ∗ *IQR*〉, where *Q1* and *Q3* represent the first and third quartile respectively and *IQR* represents the interquartile range of the data in the ANOVA cell, were winsorized to fit this interval (Wilcox, 2010). Bivariate correlations were assessed using winsorized correlation coefficient (denoted *r*_*w*_) with 10% two-sided trimming to eliminate spurious associations (Wilcox, 2010).

To compare the effects on ERSD in the present and the previous study, we first tested for homogeneity of variances in the two samples, which revealed statistically significant differences (see Supplementary Table 10). We therefore could not employ a mixed-design ANOVA and computed linear mixed models (LMMs), allowing for heterogeneous error variances in the two groups. LMMs were fitted using the R package *nlme* (function *lme*, Pinheiro, Bates, DebRoy, & Sarkar, 2014), and analyses of deviance (Type III) were performed using the package *car* (function *Anova*, Fox & Weisberg ,^2011^). LMMs were fitted separately for mu and beta ERSD as dependent variable. The fixed-effects structure was specified as containing main effects of the between-subjects factor *Setup*, and within-subjects factors *Ethnicity*, and *Treatment*, as well as all interactions (DV ~ Setup*Ethnicity*Treatment; Nieuwenhuis, Forstmann, & Wagenmakers, 2011). Regressors of factors were coded using zero-sum effect coding. A different error variance was modelled for each of the two levels of the factor Setup. To avoid anticonservative Type-I-error rates, we first fitted a maximal random effects structure, as recommended by Barr, Levy, Scheepers and Tily (2013). As LMMs with maximal random effects structure tend to be overly conservative (Matuschek, Kliegl, Vasishth, Baayen, & Bates, 2017), we furthermore fitted LMMs with reduced random effects structures and identified the most parsimonious model according to the Bayesian Information Criterion (BIC). For both mu and beta ERSD, the random effects structure identified as most parsimonious contained a random intercept per subject, as well as a random slope for *Treatment* (Supplementary Table 11)*.* With regards to significance of the fixed effects, we found only minor differences between the maximal model and the parsimoniuous model. In the main text, we therefore report the results of the parsimonious model, whereas the results of the maximal model are provided in the supplementary information (Supplementary Table 12).

### Results

#### Comparison of basic characteristics of the samples

We compared a number of variables, including trait measures, to verify equivalence of participant samples in this experiment and the one of our previous study (Riečanský et al., 2015). The two study samples did not significantly differ in age (*t*_*63*_ = −0.783, *p* = 0.436) and in proportion of males versus females (*χ*^*2*^_*1*_ = 0.001, *p* = 0.975). We also did not find any significant differences between the samples in scores of IRI, ATB, or IAT (*t* test for independent samples: all *p* values ≥ 0.195, see Supplementary Table 2).

#### Perceived bodily self-attribution

In agreement with the behavioral study, the analysis confirmed that ratings of perceived self-attribution were higher for ingroup than outgroup hands (Table 1, Ethnicity: *F*_*1,28*_**=** 25.466, *p* < 0.001, *η*^*2*^_*p*_ = 0.476, complete report of the statistical tests is presented in Supplementary Table 3). Treatment and Dimension yielded no significant effects (all *p* values for main and interaction effects ≥ 0.1), so that we averaged the data across these factors in subsequent analyses. Associations of the ratings of perceived bodily self-attribution with trait measures were weak or absent, with the exception of a moderate association between self-attribution of the outgroup hand and the ATB score (Table 1).

**Table 1:** Ratings of perceived self-attribution of the target hand and their association with trait measures

	Self-attribution (mean (SD))			Correlation coefficient (*r*_*w*_)					
	Ownership	Location	average	EC	FS	PT	PD	ATB	IAT
Ingroup hand	1.9 (1.4)	2.1 (1.4)	2.0 (1.3)	0.13	−0.02	0.12	0.07	0.24	0.11
Outgroup hand	0.9 (1.1)	1.2 (1.3)	1.1 (1.1)	−0.01	0.13	0.08	−0.13	0.37	−0.14

Note: *r*_*w*_ - 10%-winsorized correlation coefficient with the average values of perceived bodily self-attribution. EC = empathic concern, FS = fantasy, PT = perspective taking, PD = personal distress, ATB = attitudes towards blacks, IAT = implicit association test.

#### State empathy ratings

In agreement with our previous study (Riečanský et al., 2015), ratings of target-related painfulness and observer-related unpleasantness evoked by the pain videos were both slightly higher for ingroup than outgroup hands [mean (SD), painfulness ingroup vs. outgroup: 4.9 (1.5) vs. 4.8 (1.5), *t*_*28*_ = 1.74, one-tailed *p* = 0.047, *d* = 0.32; unpleasantness ingroup vs. outgroup: 3.3 (1.7) vs. 3.1 (1.7), *t*_*28*_ = 1.77, one-tailed *p* = 0.044, *d* = 0.33]. The absolute values of painfulness ratings did not differ between the studies (*F*_*1,63*_ = 2.039, *p* = 0.158, *η*^*2*^_*p*_ = 0.031). Unexpectedly, despite comparable ethnicity effects, ratings of unpleasantness were lower in the current sample compared with the previous study (*F*_*1,63*_ = 4.885, *p* = 0.031, *η*^*2*^_*p*_ = 0.072). The association of state empathy ratings with the ratings of bodily self-attribution were weak or absent (painfulness ingroup: *r*_*w*_ = −0.09, painfulness outgroup: *r*_*w*_ = −0.21, unpleasantness ingroup: *r*_*w*_ = 0.15, unpleasantness outgroup: *r*_*w*_ = 0.17).

#### EEG data

As shown in Figure 4, visual stimulation elicited suppression of oscillatory activity (i.e., ERD) in the mu (7-12 Hz) and beta (13-30 Hz) bands. ERD was induced first by the onset of the hand (time = −1,500 ms) and then by the onset of the treatment action, i.e., needle approaching and injecting the hand or cotton swab approaching and touching the hand (time = 0 ms). After the needle or swab had reached their final positions (time = 1,500 ms), ERDs gradually decreased. Given this pattern of oscillatory dynamics and in agreement with our earlier study (Riecansky et al., 2015), we separately analyzed neural responses related to the dynamic perception of the two treatments (300-1,500 ms, time window 1) and to their final static endpoint (1,800-3,000 ms, time window 2).

**Fig 5.**
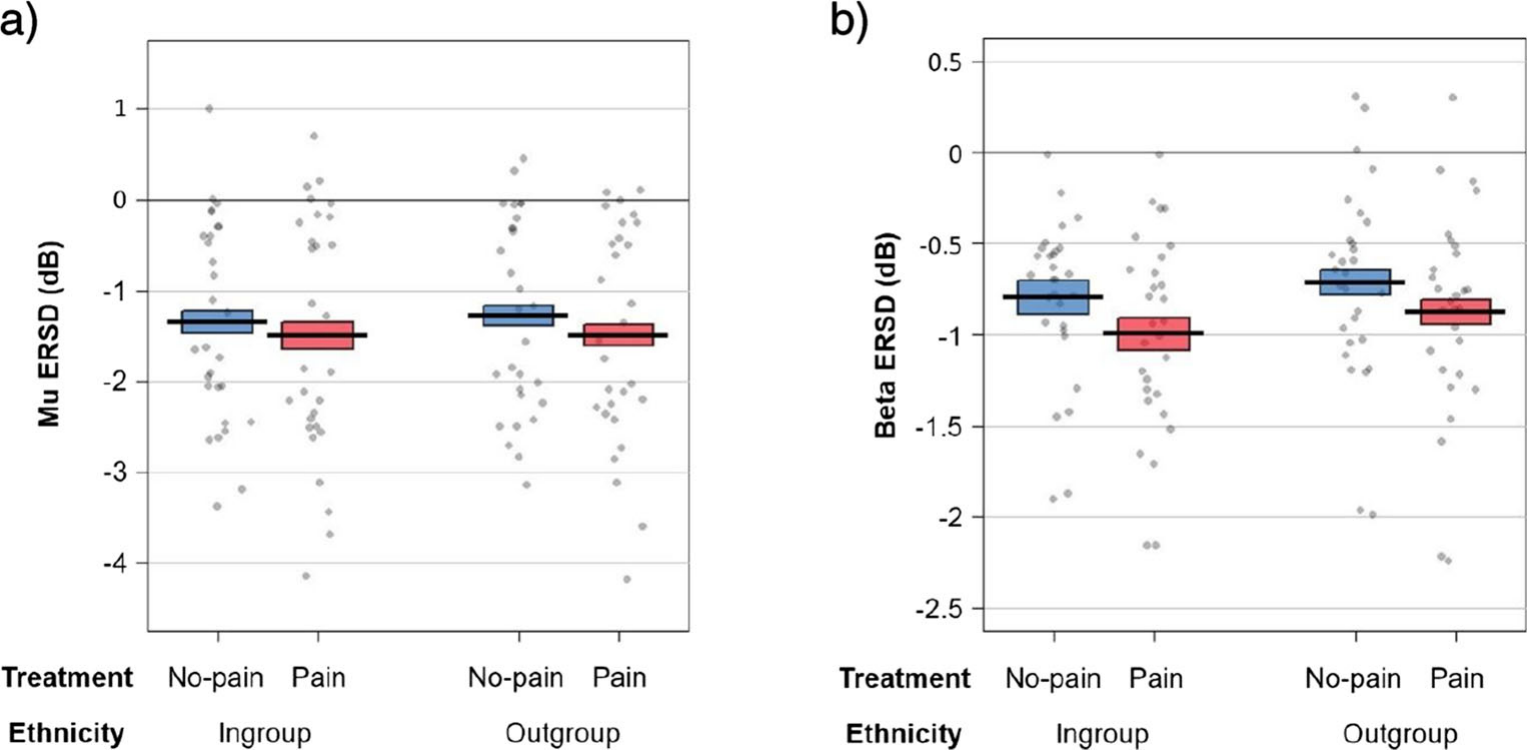
Mean mu ERSD (a) and beta ERSD (b) in each experimental condition in time window 300-1,500 ms across the ROIs. Horizontal bars: group means, boxes: 95% within-subject confidence intervals of the mean corrected for between-subject error variability (Morey, 2008). Circles: values of individual participants. Note the different scales for mu and beta ERSD. Plots were created using the function *pirateplot* of the R-package *yarrr* (Phillips, 2017)

Mu ERD in time window 1 (during “dynamic perception”) was significantly stronger during observation of painful needle injections compared with nonpainful touch by the cotton swab (Treatment: *F*_*1,28*_**=** 6.082, *p* = 0.020, *η*^*2*^_*p*_ = 0.178; Figure 5a; results in Supplementary Table 4a). All effects involving the factors Ethnicity or Hemisphere were not significant (all *p* values for main and interaction effects ≥ 0.479). In time window 2 (viewing of the “static endpoint”), the significant effect of Treatment was still present (*F*_*1,28*_**=** 8.518, *p* = 0.007, *η*^*2*^_*p*_ = 0.233; Supplementary Figure 1a), whereas the other main and interaction effects were not significant (all *p* values ≥ 0.087; results in Supplementary Table 4b).

Beta ERD also was significantly stronger during observation of pain videos compared with no-pain videos (time window 1, Treatment: *F*_*1,28*_**=** 13.882, *p* < 0.001, *η*^*2*^_*p*_ = 0.331; Supplementary Table 5a; Figure 5b). Irrespective of the treatment, beta ERD was stronger for ingroup than outgroup hands (Ethnicity: *F*_*1,28*_**=** 5.543, *p* = 0.026, *η*^*2*^_*p*_ = 0.165; Ethnicity x Treatment: *p* = 0.614). All effects involving the factor Hemisphere were not significant (all *p* values ≥ 0.305). In time window 2, the significant effect of Treatment persisted (*F*_*1,28*_**=** 9.737, *p* = 0.004, *η*^*2*^_*p*_ = 0.258, Supplementary Table 5b; Supplementary Figure 1b), but all other effects were not significant (all *p* values ≥ 0.141).

Additional analyses related to perception of the hands prior to the onset of intervention (“hand only” period, −1,200 to −300 ms) revealed a significant effect of Ethnicity in the beta band (a stronger ERD for ingroup than for outgroup hands, *F*_*1,28*_ = 6.966, *p* = 0.013, *η*^*2*^_*p*_ = 0.199) but not in the mu band (details are provided in Supplementary Table 6).

As an index of empathy-related neural activation, we next calculated the difference in the oscillatory activity elicited by pain versus no-pain videos (ERSD_E_, note that more negative values indicate a stronger ERD for pain vs. no-pain conditions and thus stronger empathy-related neural responses). For ingroup hands, higher intensity of perceived self-attribution of the target hand was moderately associated with more negative ERSD_E_ in time window 1 but later disappeared (Table 2). No statistically significant associations were found for outgroup hands, but this result must be considered with caution due to weak self-attribution of the outgroup hand. Associations between ERSD and state empathy ratings, as well as ERSD and trait measures are provided in the supplementary information (Supplementary Tables 7-9).

**Table 2:** Associations between perceived bodily self-attribution of the target hand and empathy-related neural activations (ERSD_E_)

	Mu (7-12 Hz)		Beta (13-30 Hz)	
	TW1	TW2	TW1	TW2
Ingroup hand	-.34*	.01	-.41*	-.01
Outgroup hand	-.10	-.13	.18	-.06

Data represent 10% winsorized correlation coefficient, TW1 – time window 1 (300–1,500 ms), TW2 – time window 2 (1,800–3,000 ms). *p < 0.05 (one-tailed, uncorrected)

Finally, we assessed whether “empathic” neural responses, i.e., the effects of Treatment, differed between the overlap setup (current study) and the no-overlap setup (previous study). For this purpose, we could not use ANOVA, because homogeneity tests revealed significant differences in ERSD data variability between the two samples (Supplementary Table 10). Therefore, linear mixed models (LMMs) were calculated (for details of the model, see Section 3.1.6). For mu ERSD (Table 3a), the analysis revealed a statistically significant interaction between Setup and Treatment (*χ*^*2*^_*1*_ = 6.935, *p* = 0.008), reflecting the fact that empathy-related mu ERD was stronger in the overlap setup than in the no-overlap setup. For beta ERSD (Table 3b), the LMM yielded a significant main effect of Treatment (*χ*^*2*^_*1*_ = 13.368, *p* < 0.001) and the interaction between Setup and Treatment approached statistical significance (*χ*^*2*^_*1*_ = 3.414, *p* = 0.065). In contrast to the results from the no-overlap sample alone (Riecansky et al. 2015), when the data from both samples were included, the interaction between Ethnicity and Treatment was not statistically significant. The LMM analysis, however, did not confirm that this interaction was significantly influenced by presentation Setup (i.e., a 3-way interaction between Setup, Ethnicity, and Treatment was not statistically significant).

**Table 3:** Linear mixed model analysis of the experimental effects on mu and beta ERSD. Dependent variable: mean ERSD (300-1,500 ms)

Fixed effects	Parameter (SE)	*χ*^*2*^_*(df=1)*_	*p*
a) Mu band (7-12 Hz)			
Intercept	-1.415 (0.224)	39.880	<0.001
Setup	0.007 (0.224)	0.001	0.974
Ethnicity	0.044 (0.016)	7.140	0.008
Treatment	-0.023 (0.026)	0.825	0.364
Setup*Ethnicity	-0.024 (0.016)	2.155	0.142
Setup*Treatment	-0.068 (0.026)	6.935	0.008
Ethnicity*Treatment	-0.013 (0.016)	0.644	0.422
Setup*Ethnicity*Treatment	-0.004 (0.016)	0.067	0.796
		Correlations	
Random effects	SD	Interc.	Treat.
Intercept	1.79	1.000	0.605
Treatment	0.16	0.605	1.000
Error Term (SD)			
in no-overlap sample	0.24		
in overlap sample	0.28		
b) Beta band (13-30 Hz)			
Intercept	−0.867 (0.083)	109.849	<0.001
Setup	0.009 (0.083)	0.013	0.911
Ethnicity	0.047 (0.012)	14.827	<0.001
Treatment	−0.062 (0.017)	13.368	<0.001
Setup*Ethnicity	0.013 (0.012)	1.198	0.274
Setup*Treatment	−0.031 (0.017)	3.414	0.065
Ethnicity*Treatment	0.022 (0.012)	3.285	0.070
Setup*Ethnicity*Treatment	−0.010 (0.012)	0.700	0.403
		Correlations	
Random effects	SD	Interc.	Treat.
Intercept	0.66	1.000	0.525
Treatment	0.09	0.525	1.000
Error Term (SD)			
in no-overlap sample	0.18		
in overlap sample	0.21		

Note: Random effects: Random intercept and slope of Treatment per subject. Significance tests are analyses of deviance, not t-tests of regression parameters. Factor level coding: Setup: no-overlap = −1, overlap = 1, Ethnicity: ingroup = −1, outgroup = 1, Treatment: nonpainful = −1, painful = 1.

## Discussion

This study explored, using EEG, whether weakening the bodily boundaries between the self and a target increases sensorimotor responses to the pain of that target. We also investigated whether this manipulation could possibly reduce ethnicity bias in sensorimotor activations to others' pain. We found that observing painful as opposed to nonpainful treatments of hands elicited stronger suppression of the oscillatory activity (i.e., ERD) in the mu and the beta bands over the sensorimotor cortex, and this empathy-related activation was increased by presenting stimuli in a way that weakened the bodily boundaries between the participant and the targets. Furthermore, empathy-related responses of the mu and beta rhythms were stronger in participants who reported stronger bodily self-attribution of the target hand. Finally, an ethnicity bias in empathy-related neural responses previously identified in the beta band was absent in the conditions of enhanced self-attribution of the target hand. Overall, this indicates that changes in bodily self-other distinction affect empathy-related brain activations. We will discuss these findings and their implications in more detail now.

The behavioral data of both experiments consistently suggest that our manipulation of stimulus presentation was effective and elicited the expected effects. Compared with displaying the hands on a screen in front of the participants, the overlap display significantly increased perceived self-attribution of the target hand. The absolute values of the ratings suggest that the evoked bodily perceptions were relatively weak though, which can be explained by the fact that our method predominantly relied on visual signals and did not involve congruent multimodal visuo-tactile or visuo-motor stimulation such as in the rubber hand illusion (which are known to elicit more intense illusions of body-ownership, for review, see Kilteni et al. 2015). Perceived bodily self-attribution was overall stronger with the ethnic ingroup than with outgroup targets, but the increase in self-attribution due to the overlap projection was similar for both ethnicities. This is partially in line with findings that visual features of the artificial hand may affect the rubber-hand illusion. For instance, Farmer et al. (2012) reported that rubber-hand illusion in white participants was stronger when using a rubber white hand compared with a rubber dark hand (but see Farmer et al. 2014; Maister et al. 2013). Because the ingroup preference in the illusory body-ownership was not reported in studies using immersive virtual reality, which evokes an intense ownership illusion (Banakou et al., 2016; Hasler et al., 2017; Peck et al., 2013), this intergroup bias seems to be a function of illusion strength, which in turn depends on the method used to elicit the illusion.

In addition to bodily self-awareness, sensorimotor EEG oscillations were also sensitive to our experimental manipulations. Previous studies reported that inducing illusory body (or hand) ownership elicited suppression of the central mu and beta rhythms (Evans & Blanke, 2013; Faivre et al., 2017; Lenggenhager, Halje, & Blanke, 2011; Rao & Kayser, 2017). Our results go beyond these earlier studies in showing that the intensity of perceived bodily self-attribution is also related to the strength of empathy-related activations, i.e., increased mu/beta ERD when observing painful compared with nonpainful events. Functional neuroimaging studies have demonstrated that several cortical regions that show activity modulation with bodily illusions also show activity changes in association with the strength of the mu and beta rhythms (Arnstein, Cui, Keysers, Maurits, & Gazzola, 2011; Braadbaart, Williams, & Waiter 2013; Ehrsson, Holmes, & Passingham 2005; Ehrsson, Spence, & Passingham 2004; Tsakiris, Hesse, Boy, Haggard, & Fink 2007; Yin, Liu, & Ding 2016). Our findings fit with observations that many of these brain areas (including inferior parietal, precentral, premotor, or insular cortex) are more active when seeing pain compared to non-painful or neutral stimuli (Lamm et al. 2011). It also has been reported that manipulations of bodily self-awareness influence the processing of self-directed pain stimuli (Hänsel, Lenggenhager, von Känel, Curatolo, & Blanke 2011; Romano, Llobera, & Blanke 2016; Romano, Pfeiffer, Maravita, & Blanke 2014). Our study shows that such manipulations also affect neural responses to painful stimulation of body parts of other people.

The findings of our study are in agreement with the growing knowledge on the representation of peripersonal space (i.e., the space immediately surrounding the body) and its emerging role in social affect and cognition. Peripersonal space is processed by parietal and frontal multimodal neurons, which contain congruent body-centered somatosensory and visual (or auditory) receptive fields (for review, see di Pellegrino & Làdavas, 2015; Graziano & Cooke 2006). Many such neurons are strongly activated when observing objects approaching their tactile receptive fields as well as during defensive movements to approaching noxious objects, indicating the role of peripersonal space representations in protecting the body from injuries (Graziano et al. 1997). Numerous findings suggest that peripersonal space is plastic and can be extended to include other individuals (Brozzoli et al. 2013; Cardellicchio et al. 2013; Costantini et al. 2011; Ishida et al. 2010; Teneggi et al. 2013; Thomas et al. 2006, for review, see Brozzoli et al. 2014; de Vignemont, 2014). Furthermore, peripersonal space undergoes flexible changes with changing possibilities of bodily interactions. For instance, holding and using a tool to reach for objects extends the peripersonal space to involve the space around the tool (for a recent review, see Martel et al. 2016). Rossetti et al. (2015) reported that humans reacted with increased autonomic arousal when harmful objects appeared in the vicinity of the (actively used) tool, indicating that body's protective zone had enlarged to include the space around the tool. Similar effects result from inducing hand ownership illusion: threatening or injuring a self-attributed fake hand evokes autonomic and motor activation (Armel & Ramachandran 2003; Ehrsson, Wiech, Weiskopf, Dolan, & Passingham, 2007; Fusaro, Tieri, & Aglioti, 2016; González-Franco, Peck, Rodríguez-Fornells, & Slater, 2014; Lloyd, Morrison, & Roberts 2006). We thus have good reason to assume that the peripersonal space underwent similar shifts also in our study, i.e., the increased bodily self-attribution of the target hand resulted in a remapping of the defensive peripersonal space to include the target hand, which in turn enhanced sensorimotor responses to observed painful versus neutral events. However, this interpretation needs to be corroborated by explicit measures of peripersonal space, which we did not include in our study design.

Numerous studies demonstrated that observing painful stimulations delivered to the hand of a model activates motor processes. It is still debated, however, which specific neural processes are induced in such situations. In particular, research using transcranial magnetic stimulation of primary motor cortex brought contradictory results, with some studies indicating decreased cortical excitability, which would be a sign of movement inhibition (Avenanti et al. 2005, 2006, 2009b; Fecteau et al. 2008), whereas other studies showed increased cortical excitability, and hence signs of movement facilitation (De Coster et al. 2014; de Guzman, Bird, Banissy, & Catmur, 2016; Fitzgibbon et al. 2012). These discrepancies have been discussed from diverse viewpoints, including the perceived hand ownership and control (Bucchioni et al. 2016; De Coster et al. 2014; de Guzman et al. 2016), the physical distance of the stimulated hand from the observer (Mahayana et al. 2014), the viewing perspective (Bucchioni et al. 2016), ingroup-outgroup distinction (Avenanti et al. 2010), or personality traits (Avenanti et al. 2009a; Fecteau et al. 2008). Because mu/beta ERD is associated with increased excitability of primary motor cortex (Takemi, Masakado, Liu, & Ushiba, 2013), our results indicate that seeing a painful stimulation is associated with a facilitation of movements probably indicating an increased readiness for a defensive motor reaction or escape (Galang et al. 2017; Morrison et al. 2007), which is, moreover, positively related to the perceived self-attribution of the target hand (Fitzgibbon et al. 2012). This is consistent with the findings of González-Franco et al. (2014) who used immersive virtual reality to induce illusory ownership of the hand of an avatar. The authors found that seeing injuries of the virtual hand compared with the neutral images evoked stronger mu ERD and a higher amplitude of the readiness potential, thus indicating movement preparation (see also Galang et al. 2017). Moreover, other studies using similar paradigms reported an urge of participants to retreat the hand if a virtual self-attributed hand is threatened (Ehrsson et al. 2007). The absent lateralization of ERD does not rule out such an explanation since unilateral limb movements can be associated with bilateral ERD (Crone et al. 1998). Similarly, ERD is bilateral during observation of unilateral hand movements (Avanzini et al. 2012). Unfortunately, we cannot claim with certainty a conscious movement tendency also occurred in our study since we had not collected data on perceived action tendencies of the study participants. On the other hand, as the amplitude of the central rhythms correlates with activity in many cortical areas (Arnstein et al., 2011; Braadbaart et al., 2013; Yin et al., 2016), it cannot be ruled out that the increased mu/beta ERD to painful versus neutral stimuli was mainly related to neuronal processing outside of primary motor cortex (such as in the parietal, premotor, or insular cortex). These aspects and the precise neurophysiological mechanisms of the effects we observed therefore need to be addressed in future studies.

Personal identity is tightly linked with identification with a social group (e.g., ethnic, religious, or political), which shapes perceptions, emotions, thoughts, judgments, decisions, and actions (for review, see Ellemers et al., 2002). Social psychology research has revealed a general tendency of human beings to evaluate members of one’s own group more positively than outgroup members (for review, see Hewstone et al., 2002). Such an ingroup favoritism also concerns empathy: people empathize more strongly with ingroup members and show more prosocial behavior towards them (for review, see Cikara et al., 2011; Eisenberg et al., 2010). In recent years, a number of studies have documented that brain responses are higher when observing pain inflicted in ethnic ingroup members (for review, see Cikara & Van Bavel, 2014; Han, 2018; Molenberghs, 2013, Vollberg & Cikara, 2018). Several previous studies reported that self-attribution of an outgroup body can reduce ingroup bias in implicit judgements and attitudes (for review, see Maister et al., 2015). Our data show that weakening bodily self-other boundaries increases empathy-related sensorimotor neural responses to both ingroup and outgroup targets. Although we expected that enhancing bodily overlap with the other persons’ hands will decrease an ingroup bias in empathy-related beta-band responses, our data are not conclusive in this regard. Ethnicity bias in beta-band empathy-related activations was only present in the no-overlap presentation but was absent in the overlap setup. Conversely, a direct comparison of the activations between the setups did not confirm a significant effect on the magnitude of the bias. Thus, further investigations using manipulations of bodily self-other boundaries are warranted in the light of a current debate on the role of empathy in improving intergroup relations, social justice, and human rights (Hoffman, 2014; Jackson, Eugène, & Tremblay, 2015; Levy et al., 2016; Vanman, 2016).

## Electronic supplementary material

ESM 1 (DOCX 178 kb)
