# Supplementary material for: Increasing self-other bodily overlap increases sensorimotor resonance to others’ pain
Source: Cogn Affect Behav Neurosci. 2019 Jun 12;20(1):19–33. doi: 10.3758/s13415-019-00724-0 (PMC7012796; doi:10.3758/s13415-019-00724-0)

**SUPPLEMENTARY INFORMATION**

Abbreviation used: ANOVA - analysis of variance, BS – between subjects, DV - dependent variable, IV - independent variable, RM - repeated measures, WS - within subjects.

**Supplementary table 1.** Behavioral experiment: GLM analysis of the experimental effects on perceived bodily self-attribution

**a)** **Model: 2×2×2×3** mixed between-within subjects ANOVA, BS IV: Setup (2 levels), WS IVs: Ethnicity (2 levels), Treatment (2 levels), Dimension (3 levels: Ownership, Location and Agency), DV: rating of perceived bodily self-attribution

Effect ANOVA

Setup *F_1,40_* = 9.443, *p* = 0.004, *η²_p_* = 0.191

Setup*Ethnicity *F_1,40_* = 0.209, *p* = 0.650, *η²_p_* = 0.005

Setup*Treatment *F_1,40_* = 0.056, *p* = 0.814, *η²_p_* = 0.001

Setup*Dimension *F_2,80_* = 2.008, *p* = 0.164, *η²_p_* = 0.048

Setup*Ethnicity*Treatment *F_1,40_* = 0.264, *p* = 0.610, *η²_p_* = 0.007

Setup*Ethnicity*Dimension *F_2,80_* = 1.447, *p* = 0.241, *η²_p_* = 0.035

Setup*Treatment*Dimension *F_2,80_* = 0.156, *p* = 0.856, *η²_p_* = 0.027

Setup*Ethnicity*Treatment*Dimension *F_2,80_* = 0.286, *p* = 0.752, *η²_p_* = 0.007

Ethnicity *F_1,40_* = 13.193, *p* = 0.001, *η²_p_* = 0.248

Ethnicity*Treatment *F_1,40_* = 1.504, *p* = 0.227, *η²_p_* = 0.036

Ethnicity*Dimension ^a)^ *F_2,80_* = 7.714, *p* = 0.001, *η²_p_* = 0.162

Ethnicity*Treatment*Dimension *F_2,80_* = 1.092, *p* = 0.340, *η²_p_* = 0.027

Treatment *F_1,40_* = 2.401, *p* = 0.129, *η²_p_* = 0.057

Dimension *F_2,80_* = 38.178, *p* < 0.001, *η²_p_* = 0.488

Treatment*Dimension *F_2,80_* = 0.728, *p* = 0.486, *η²_p_* = 0.018

^a)^ Ethnicity effects for each category of Dimension: (1) *Ownership: F_1,40_* = 1.98, *p* < 0.001; (2) *Location: F_1,40_*= 10.10*, p* = 0.003; (3) *Agency: F_1,40_* = .20*, p* = 0.689

**b) Model: 2×2×2×2** mixed between-within subjects ANOVA, BS IV: Setup (2 levels), WS IVs: Ethnicity (2 levels), Treatment (2 levels), Dimension (2 levels: Ownership and Location), DV: rating of bodily self-attribution

Effect ANOVA

Setup *F_1,40_* = 8.724, *p* = 0.005, *η²_p_* = 0.179

Setup*Ethnicity *F_1,40_* = 0.522, *p* = 0.474, *η²_p_* = 0.013

Setup*Treatment *F_1,40_* = 0.005, *p* = 0.946, *η²_p_* = 0.001

Setup*Dimension *F_1,40_* = 0.237, *p* = 0.629, *η²_p_* = 0.006

Setup*Ethnicity*Treatment *F_1,40_* = 0.254, *p* = 0.617, *η²_p_* = 0.006

Setup*Ethnicity*Dimension *F_1,40_* = 1.639, *p* = 0.208, *η²_p_* = 0.039

Setup*Treatment*Dimension *F_1,40_* = 0.172, *p* = 0.681, *η²_p_* = 0.004

Setup*Ethnicity*Treatment*Dimension *F_1,40_* = 0.421, *p* = 0.520, *η²_p_* = 0.010

Ethnicity *F_1,40_* = 15.102, *p* < 0.001, *η²_p_* = 0.274

Ethnicity*Treatment *F_1,40_* = 1.605, *p* = 0.213, *η²_p_* = 0.039

Ethnicity*Dimension *F_1,40_* = 0.095, *p* = 0.759, *η²_p_* = 0.002

Ethnicity*Treatment*Dimension *F_1,40_* = 0.689, *p* = 0.412, *η²_p_* = 0.017

Treatment *F_1,40_* = 2.022, *p* = 0.163, *η²_p_* = 0.048

Dimension *F_1,40_* = 0.387, *p* = 0.537, *η²_p_* = 0.010

Treatment*Dimension *F_1,40_* = 0.799, *p* = 0.377, *η²_p_* = 0.020

**Supplementary table 2.** EEG experiment: descriptive statistics of participants’ trait measures and comparison with the control sample (from Riečanský et al., 2015).

Control sample Current sample

mean (SD) mean (SD) *t*-test for indep. samples

Empathic concern 15.3 (1.6) 15.2 (2.8) *t_63_ =* 0.214, *p =* 0.831

Fantasy 14.4 (2.2) 14.9 (2.8) *t_63_ =* -0.803, *p =* 0.425

Perspective taking 15.0 (2.2) 15.3 (3.5) *t_63_ =* -0.357, *p =* 0.723

Personal distress 12.1 (2.4) 11.2 (3.2) *t_63_ =* 1.310, *p =* 0.195

Attitudes towards blacks 6.0 (0.4) 6.1 (0.5) *t_63_ =* -0.659, *p =* 0.512

Implicit association test 0.39 (0.47) 0.45 (0.48) *t_63_ =* -0.507, *p =* 0.614

**Supplementary table 3.** EEG experiment: GLM analysis of the experimental effects on perceived bodily self-attribution

Model: 2×2×2 within subjects ANOVA, WS IVs: Ethnicity, Treatment, Dimension, DV: ratings of perceived bodily self-attribution

Effect ANOVA

Ethnicity *F_1,28_* **=** 25.466, *p <* 0.001, *η²_p_* = 0.476

Treatment *F_1,28_* **=** 2.892, *p* = 0.100, *η²_p_* = 0.094

Dimension *F_1,28_* **=** 2.451, *p* = 0.129, *η²_p_* = 0.080

Ethnicity*Treatment *F_1,28_* **=** 0.544, *p* = 0.467, *η²_p_* = 0.019

Ethnicity*Dimension *F_1,28_* **=** 0.433, *p* = 0.516, *η²_p_* = 0.015

Treatment*Dimension *F_1,28_* **=** 0.941, *p* = 0.340, *η²_p_* = 0.033

Ethnicity*Treatment*Dimension *F_1,28_* **=** 0.262, *p* = 0.613, *η²_p_* = 0.009

**Supplementary table 4.** GLM analysis of the experimental effects on mu ERSD

Model: 2×2×2 within subjects ANOVA; WS IVs: Ethnicity, Treatment, Hemisphere; DV: mean mu ERSD (7-12 Hz) within the given time window

**a) Time window 1 (300-1500 ms)**

Effect ANOVA

Ethnicity *F_1,28_* **=** 0.515, *p* = 0.479, *η²_p_* = 0.018

Treatment *F_1,28_* **=** 6.082, *p* = 0.020, *η²_p_* = 0.178

Hemisphere *F_1,28_* **=** 0.494, *p* = 0.488, *η²_p_* = 0.017

Ethnicity*Treatment *F_1,28_* **=** 0.514, *p* = 0.479, *η²_p_* = 0.018

Ethnicity*Hemisphere *F_1,28_* **=** 0.016, *p* = 0.900, *η²_p_* = 0.001

Treatment*Hemisphere *F_1,28_* **=** 0.353, *p* = 0.557, *η²_p_* = 0.012

Ethnicity*Treatment*Hemisphere *F_1,28_* **=** 0.003, *p* = 0.958, *η²_p_* < 0.001

**b) Time window 2 (1800-3000 ms)**

Effect ANOVA

Ethnicity *F_1,28_* **=** 0.001, *p* = 0.978, *η²_p_* < 0.001

Treatment *F_1,28_* **=** 8.518, *p* = 0.007, *η²_p_* = 0.233

Hemisphere *F_1,28_* **=** 1.039, *p* = 0.317, *η²_p_* = 0.036

Ethnicity*Treatment *F_1,28_* **=** 2.145, *p* = 0.154, *η²_p_* = 0.071

Ethnicity*Hemisphere *F_1,28_* **=** 1.218, *p* = 0.279, *η²_p_* = 0.042

Treatment*Hemisphere *F_1,28_* **=** 0.191, *p* = 0.666, *η²_p_* = 0.007

Ethnicity*Treatment*Hemisphere *F_1,28_* **=** 3.281, *p* = 0.081, *η²_p_* = 0.105

**Supplementary table 5.** GLM analysis of the experimental effects on beta ERSD

Model: 2×2×2 within subjects ANOVA; WS IVs: Ethnicity, Treatment, Hemisphere; DV: mean beta ERSD (13-30 Hz) within the given time window

**a) Time window 1 (300-1500 ms)**

Effect ANOVA

Ethnicity *F_1,28_* **=** 5.543, *p* = 0.026, *η²_p_* = 0.165

Treatment *F_1,28_* **=** 13.882, *p* < 0.001, *η²_p_* = 0.331

Hemisphere *F_1,28_* **=** 0.462, *p* = 0.502, *η²_p_* = 0.016

Ethnicity*Treatment *F_1,28_* **=** 0.260, *p* = 0.614, *η²_p_* = 0.009

Ethnicity*Hemisphere *F_1,28_* **=** 1.093, *p* = 0.305, *η²_p_* = 0.038

Treatment*Hemisphere *F_1,28_* **=** 0.421, *p* = 0.552, *η²_p_* = 0.015

Ethnicity*Treatment*Hemisphere *F_1,28_* **=** 0.086, *p* = 0.771, *η²_p_* = 0.003

**b) Time window 2 (1800-3000 ms)**

Effect ANOVA

Ethnicity *F_1,28_* **=** 2.297, *p* = 0.141, *η²_p_* = 0.076

Treatment *F_1,28_* **=** 9.737, *p* = 0.004, *η²_p_* = 0.258

Hemisphere *F_1,28_* **=** 2.505, *p* = 0.125, *η²_p_* = 0.082

Ethnicity*Treatment *F_1,28_* **=** 0.549, *p* = 0.465, *η²_p_* = 0.019

Ethnicity*Hemisphere *F_1,28_* **=** 0.016, *p* = 0.899, *η²_p_* = 0.001

Treatment*Hemisphere *F_1,28_* **=** 0.850, *p* = 0.364, *η²_p_* = 0.029

Ethnicity*Treatment*Hemisphere *F_1,28_* **=** 0.050, *p* = 0.825, *η²_p_* = 0.002

**Supplementary table 6.** GLM analysis of the experimental effects on ERSD prior to treatment onset (observation of hands, -1200 to -300 ms)

Model: 2×2 within subjects ANOVA; WS IVs: Ethnicity, Hemisphere; DV: mean ERSD

**a) Mu ERSD (7-12 Hz)**

Effect ANOVA

Ethnicity *F_1,28_* = 0.006, *p* = 0.937, *η²_p_* < 0.001

Hemisphere *F_1,28_* = 1.015, *p* = 0.322, *η²_p_* = 0.035

Ethnicity*Hemisphere *F_1,28_* = 0.383, *p* = 0.541, *η²_p_* = 0.013

**b) Beta ERSD (13-30 Hz)**

Effect ANOVA

Ethnicity ^a)^ *F_1,28_* = 6.966, *p* = 0.013, *η²_p_* = 0.199

Hemisphere *F_1,28_* = 0.597, *p* = 0.446, *η²_p_* = 0.021

Ethnicity*Hemisphere *F_1,28_* = 0.362, *p* = 0.552, *η²_p_* = 0.013

^a)^ Mean ± SEM: ingroup hands = -0.512 ± 0.062 dB, outgroup hands = -0.427 ± 0.064 dB

**Supplementary table** **7**: Association between mu and beta ERSD and state empathy ratings

Data: 10% winsorized correlation coefficient. TW1: 300-1500 ms, TW2: 1800-3000 ms, ERSD_P_: ERSD in pain conditions, ERSD_E_: ERSD in pain conditions vs. ERSD in no-pain conditions

Painfulness Unpleasantness

TW1 TW2 TW1 TW2

ingroup target

mu ERSD_P_ 0.05 0.05 -0.09 -0.06

ERSD_E_ 0.23 0.02 0.32 0.25

beta ERSD_P_ 0.12 0.09 0.03 0.07

ERSD_E_ 0.28 0.25 0.06 0.26

outgroup target

mu ERSD_P_ -0.04 -0.10 -0.12 -0.18

ERSD_E_ -0.05 0.07 -0.01 0.09

beta ERSD_P_ -0.09 -0.11 -0.03 -0.14

ERSD_E_ 0.07 0.14 0.20 0.08

**Supplementary table 8: GLM analysis of the effects of trait measures on mu ERSD**

Model: 2×2 within subjects ANCOVA; WS IVs: Ethnicity, Treatment, DV: mean mu ERSD (7-12 Hz) in Time window 1 (300 - 1500 ms). Covariates: Trait measures (specified below).

*a) Empathic Concern*

Effect Test statistics Significance Effect size

Empathic Concern *F_1,27_* **=** 0.107 *p* = 0.746 *η²_p_* = 0.004

*Ethnicity *F_1,27_* **=** 0.068 *p* = 0.796 *η²_p_* = 0.003

*Treatment *F_1,27_* **=** 1.047 *p* = 0.315 *η²_p_* = 0.037

*Ethnicity*Treatment *F_1,27_* **=** 1.913 *p* = 0.178 *η²_p_* = 0.066

*b) Fantasy*

Effect Test statistics Significance Effect size

Fantasy *F_1,27_* **=** 1.357 *p* = 0.254 *η²_p_* = 0.048

*Ethnicity *F_1,27_* **=** 1.934 *p* = 0.176 *η²_p_* = 0.067

*Treatment *F_1,27_* **=** 0.084 *p* = 0.774 *η²_p_* = 0.003

*Ethnicity*Treatment *F_1,27_* **=** 0.003 *p* = 0.955 *η²_p_* < 0.001

*c) Perspective Taking*

Effect Test statistics Significance Effect size

Perspective Taking *F_1,27_* **=** 1.330 *p* = 0.259 *η²_p_* = 0.047

*Ethnicity *F_1,27_* **=** 1.066 *p* = 0.311 *η²_p_* = 0.038

*Treatment *F_1,27_* **=** 0.650 *p* = 0.427 *η²_p_* = 0.023

*Ethnicity*Treatment *F_1,27_* **=** 0.394 *p* = 0.535 *η²_p_* = 0.014

*d) Personal Distress*

Effect Test statistics Significance Effect size

Personal Distress *F_1,27_* **=** 2.831 *p* = 0.104 *η²_p_* = 0.095

*Ethnicity *F_1,27_* **=** 0.916 *p* = 0.347 *η²_p_* = 0.033

*Treatment *F_1,27_* **=** 1.294 *p* = 0.265 *η²_p_* = 0.046

*Ethnicity*Treatment *F_1,27_* **=** 0.202 *p* = 0.656 *η²_p_* = 0.007

*e) Attitudes towards blacks*

Effect Test statistics Significance Effect size

Attitudes towards blacks *F_1,27_* **=** 0.016 *p* = 0.899 *η²_p_* = 0.001

*Ethnicity *F_1,27_* **=** 0.563 *p* = 0.459 *η²_p_* = 0.020

*Treatment *F_1,27_* **=** 1.399 *p* = 0.247 *η²_p_* = 0.049

*Ethnicity*Treatment *F_1,27_* **=** 0.943 *p* = 0.340 *η²_p_* = 0.034

*f) Implicit Association Test*

Effect Test statistics Significance Effect size

Implicit Association Test *F_1,27_* **=** 0.782 *p* = 0.384 *η²_p_* = 0.028

*Ethnicity *F_1,27_* **=** 0.783 *p* = 0.384 *η²_p_* = 0.028

*Treatment *F_1,27_* **=** 0.196 *p* = 0.661 *η²_p_* = 0.007

*Ethnicity*Treatment *F_1,27_* **=** 4.082 *p* = 0.053 *η²_p_* = 0.131

**Supplementary table 9: GLM analysis of the effects of trait measures on beta ERSD**

Model: 2×2 within subjects ANCOVA; WS IVs: Ethnicity, Treatment, DV: mean beta ERSD (7-12 Hz) in Time window 1 (300 - 1500 ms). Covariates: Trait measures (specified below).

*a) Empathic Concern*

Effect Test statistics Significance Effect size

Empathic Concern *F_1,27_* **=** 0.618 *p* = 0.439 *η²_p_* = 0.022

*Ethnicity *F_1,27_* **=** 0.557 *p* = 0.462 *η²_p_* = 0.020

*Treatment *F_1,27_* **=** 0.172 *p* = 0.681 *η²_p_* = 0.006

*Ethnicity*Treatment *F_1,27_* **=** 3.175 *p* = 0.086 *η²_p_* = 0.105

*b) Fantasy*

Effect Test statistics Significance Effect size

Fantasy *F_1,27_* **=** 0.009 *p* = 0.925 *η²_p_* < 0.001

*Ethnicity *F_1,27_* **=** 0.487 *p* = 0.491 *η²_p_* = 0.018

*Treatment *F_1,27_* **=** 0.133 *p* = 0.718 *η²_p_* = 0.005

*Ethnicity*Treatment *F_1,27_* **=** 0.026 *p* = 0.872 *η²_p_* = 0.001

*c) Perspective Taking*

Effect Test statistics Significance Effect size

Perspective Taking *F_1,27_* **=** 1.090 *p* = 0.306 *η²_p_* = 0.039

*Ethnicity *F_1,27_* **=** 0.068 *p* = 0.796 *η²_p_* = 0.003

*Treatment *F_1,27_* **=** 5.163 *p* = 0.031 *η²_p_* = 0.161

*Ethnicity*Treatment *F_1,27_* **=** 1.294 *p* = 0.265 *η²_p_* = 0.046

*d) Personal Distress*

Effect Test statistics Significance Effect size

Personal Distress *F_1,27_* **=** 1.363 *p* = 0.253 *η²_p_* = 0.048

*Ethnicity *F_1,27_* **=** 1.738 *p* = 0.199 *η²_p_* = 0.060

*Treatment *F_1,27_* **=** 2.439 *p* = 0.130 *η²_p_* = 0.083

*Ethnicity*Treatment *F_1,27_* **=** 2.573 *p* = 0.120 *η²_p_* = 0.087

*e) Attitudes towards blacks*

Effect Test statistics Significance Effect size

Attitudes towards blacks *F_1,27_* **=** 0.602 *p* = 0.445 *η²_p_* = 0.022

*Ethnicity *F_1,27_* **=** 3.331 *p* = 0.079 *η²_p_* = 0.110

*Treatment *F_1,27_* **=** 0.338 *p* = 0.566 *η²_p_* = 0.012

*Ethnicity*Treatment *F_1,27_* **=** 0.216 *p* = 0.646 *η²_p_* = 0.008

*f) Implicit Association Test*

Effect Test statistics Significance Effect size

Implicit Association Test *F_1,27_* **=** 1.593 *p* = 0.218 *η²_p_* = 0.056

*Ethnicity *F_1,27_* **=** 0.882 *p* = 0.356 *η²_p_* = 0.032

*Treatment *F_1,27_* **=** 0.654 *p* = 0.426 *η²_p_* = 0.024

*Ethnicity*Treatment *F_1,27_* **=** 0.090 *p* = 0.766 *η²_p_* = 0.003

**Supplementary table 10: Tests of homogeneity of error variances in the non-overlap and the overlap samples**

Levene's test of homogeneity of error variances, calculated separately for each combination of the factors Ethnicity and Treatment

**a) DV: mu ERSD (7-12 Hz, 300-1500 ms)**

Factor combination *F_(1,63)_ p*

Ingroup, No-Pain 5.624 0.021

Ingroup, Pain 5.038 0.028

Outgroup, No-Pain 4.976 0.029

Outgroup, Pain 5.556 0.022

**b) DV: beta ERSD (13-30 Hz, 1800 – 3000 ms)**

Factor combination *F_(1,63)_ p*

Ingroup, No-Pain 4.935 0.030

Ingroup, Pain 2.869 0.095

Outgroup, No-Pain 2.634 0.110

Outgroup, Pain 3.357 0.072

**Supplementary table 11: Bayesian information criteria for LMM with different random effects structure**

Bayesion information criteria (BIC) for linear mixed models with different random effects structures.
DV: mean ERSD in Time window 1 (300 - 1500 ms). Fixed effects: Main effects of *Setup*, *Ethnicity* and *Treatment*, and all interactions. BIC values are missing for model variants where parameter estimation did not converge.

Random effects mu ERSD beta ERSD

Intercept only 548.88 285.86

Ethnicity - 296.90

Treatment 524.00 278.47

Ethnicity + Treatment - 286.79

Ethnicity + Treatment + Ethnicity x Treatment 549.72 301.89

**Supplementary table 12: Linear mixed model for ERSD with the maximal random effects structure**

DV: mean ERSD (300-1500 ms). Note: Random effects: Random intercept and slope of Treatment per subject. Significance tests are analyses of deviance, not t-tests of regression parameters. Factor level coding: Setup: no-overlap = -1, overlap = 1, Ethnicity: ingroup = -1, outgroup = 1, Treatment: non-painful = -1, painful = 1.

**a) Mu band (7-12 Hz)**

Fixed effects Parameter (SE) *χ^2^_(df=1)_* *p*

Intercept -1.415 (0.224) 39.866 < .001

Setup^a^ 0.007 (0.224) 0.001 0.974

Ethnicity^b^ 0.044 (0.016) 8.084 0.004

Treatment^c^  -0.023 (0.026) 0.817 0.366

Setup*Ethnicity -0.024 (0.016) 2.440 0.118

Setup*Treatment -0.068 (0.026) 6.871 0.009

Ethnicity*Treatment -0.013 (0.016) 0.560 0.454

Setup*Ethnicity*Treatment -0.004 (0.016) 0.058 0.810

Correlations

Random effect SD Int. Eth. Treat. Eth*Treat

Intercept 1.79 1.000 -0.439 0.539 0.177

Ethnicity 0.07 1.000 -0.571 0.353

Treatment 0.18 1.000 -0.225

Ethnicity*Treatment 0.09 1.000

Error Term (SD)

in no-overlap sample 0.17

in overlap sample 0.23

**b) Beta band (13-30 Hz)**

Fixed effects Parameter (SE) *χ^2^_(df=1)_* *p*

Intercept -0.867 (0.083) 109.849 < .001

Setup^a^ 0.009 (0.083) 0.013 0.911

Ethnicity^b^ 0.047 (0.012) 14.827 < .001

Treatment^c^  -0.062 (0.017) 13.368 < .001

Setup*Ethnicity 0.013 (0.012) 1.198 0.335

Setup*Treatment -0.031 (0.017) 3.414 0.066

Ethnicity*Treatment 0.022 (0.012) 3.285 0.031

Setup*Ethnicity*Treatment -0.010 (0.012) 0.700 0.318

Correlations

Random effect SD Int. Eth. Treat. Eth*Treat

Intercept 0.66 1.000 -0.002 0.409 -0.201

Ethnicity 0.09 1.000 -0.214 0.394

Treatment 0.12 1.000 -0.455

Ethnicity*Treatment 0.05 1.000

Error Term (SD)

in no-overlap sample 0.10

in overlap sample 0.14

**Supplementary figure 1:** Mean mu ERSD (a) and beta ERSD (b) in each experimental condition in time window 1800-3000 ms (observation of static picture – treatment endpoint) across the ROIs. Horizontal bars: group means, boxes: 95% within-subject confidence intervals of the mean corrected for between-subject error variability (Morey, 2008), circles: values of individual participants. Note the different scales for mu and beta ERSD. Plots were created using the function *pirateplot* of the R-package *yarrr* (Phillips, 2017).

**a) Mu ERSD (7-12 Hz)**


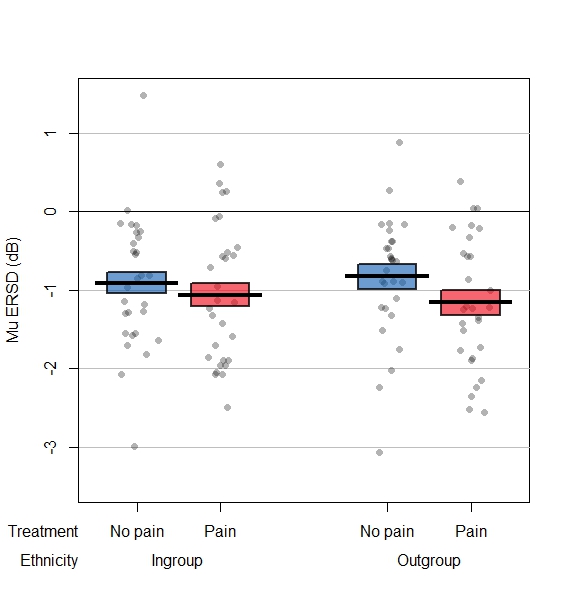


**b) Beta ERSD (13-30 Hz)**


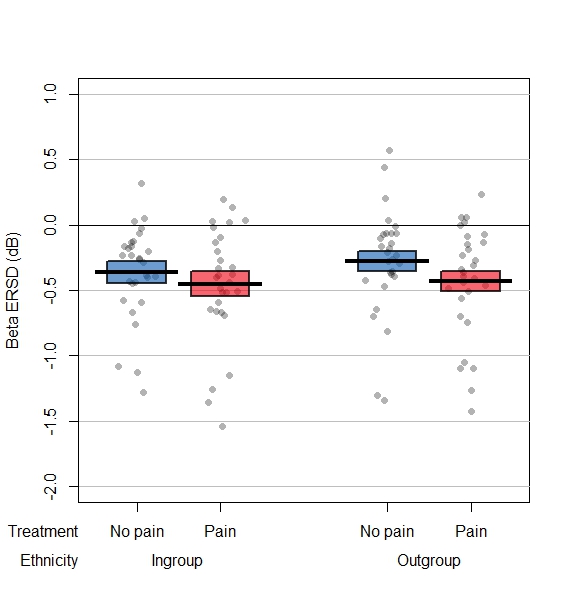

Supplement: Supplementary file 1 — (DOCX 178 kb) [file 13415_2019_724_MOESM1_ESM.docx]
